# Cognitive deficits in problematic internet use: meta-analysis of 40 studies

**DOI:** 10.1192/bjp.2019.3

**Published:** 2019-11

**Authors:** Konstantinos Ioannidis, Roxanne Hook, Anna E. Goudriaan, Simon Vlies, Naomi A. Fineberg, Jon E. Grant, Samuel R. Chamberlain

**Affiliations:** 1 Consultant Psychiatrist, Cambridge and Peterborough NHS Foundation Trust; and Honorary Visiting Fellow, Department of Psychiatry, University of Cambridge, UK; 2 Research Assistant, Department of Psychiatry, University of Cambridge, UK; 3 Professor in Addiction, Academic Medical Center, Department of Psychiatry and Amsterdam Institute for Addiction Research, University of Amsterdam; and Arkin Mental Health Care, Netherlands; 4 Foundation Doctor Year 1, Cambridge and Peterborough NHS Foundation Trust, UK; 5 Consultant Psychiatrist and Visiting Professor, Hertfordshire Partnership University NHS Foundation Trust, University of Hertfordshire; and Senior Clinical Research Fellow, University of Cambridge School of Clinical Medicine, UK; 6 Professor, Department of Psychiatry, University of Chicago, Pritzker School of Medicine, USA; 7 Department of Psychiatry, University of Cambridge; and Peterborough NHS Foundation Trust, Cambridge, UK

**Keywords:** Behavioral addiction, internet addiction, internet gaming disorder, problematic internet use, meta-analysis

## Abstract

**Background:**

Excessive use of the internet is increasingly recognised as a global public health concern. Individual studies have reported cognitive impairment in problematic internet use (PIU), but have suffered from various methodological limitations. Confirmation of cognitive deficits in PIU would support the neurobiological plausibility of this disorder.

**Aims:**

To conduct a rigorous meta-analysis of cognitive performance in PIU from case–control studies; and to assess the impact of study quality, the main type of online behaviour (for example gaming) and other parameters on the findings.

**Method:**

A systematic literature review was conducted of peer-reviewed case–controlled studies comparing cognition in people with PIU (broadly defined) with that of healthy controls. Findings were extracted and subjected to a meta-analysis where at least four publications existed for a given cognitive domain of interest.

**Results:**

The meta-analysis comprised 2922 participants across 40 studies. Compared with controls, PIU was associated with significant impairment in inhibitory control (Stroop task Hedge's *g* = 0.53 (s.e. = 0.19–0.87), stop-signal task *g* = 0.42 (s.e. = 0.17–0.66), go/no-go task *g* = 0.51 (s.e. = 0.26–0.75)), decision-making (*g* = 0.49 (s.e. = 0.28–0.70)) and working memory (*g* = 0.40 (s.e. = 0.20–0.82)). Whether or not gaming was the predominant type of online behaviour did not significantly moderate the observed cognitive effects; nor did age, gender, geographical area of reporting or the presence of comorbidities.

**Conclusions:**

PIU is associated with decrements across a range of neuropsychological domains, irrespective of geographical location, supporting its cross-cultural and biological validity. These findings also suggest a common neurobiological vulnerability across PIU behaviours, including gaming, rather than a dissimilar neurocognitive profile for internet gaming disorder.

**Declaration of interest:**

S.R.C. consults for Cambridge Cognition and Shire. K.I.’s research activities were supported by Health Education East of England Higher Training Special interest sessions. A.E.G.'s research has been funded by Innovational grant (VIDI-scheme) from ZonMW: (91713354). N.A.F. has received research support from Lundbeck, Glaxo-SmithKline, European College of Neuropsychopharmacology (ECNP), Servier, Cephalon, Astra Zeneca, Medical Research Council (UK), National Institute for Health Research, Wellcome Foundation, University of Hertfordshire, EU (FP7) and Shire. N.A.F. has received honoraria for lectures at scientific meetings from Abbott, Otsuka, Lundbeck, Servier, Astra Zeneca, Jazz pharmaceuticals, Bristol Myers Squibb, UK College of Mental Health Pharmacists and British Association for Psychopharmacology (BAP). N.A.F. has received financial support to attend scientific meetings from RANZCP, Shire, Janssen, Lundbeck, Servier, Novartis, Bristol Myers Squibb, Cephalon, International College of Obsessive-Compulsive Spectrum Disorders, International Society for Behavioral Addiction, CINP, IFMAD, ECNP, BAP, the World Health Organization and the Royal College of Psychiatrists. N.A.F. has received financial royalties for publications from Oxford University Press and payment for editorial duties from Taylor and Francis. J.E.G. reports grants from the National Center for Responsible Gaming, Forest Pharmaceuticals, Takeda, Brainsway, and Roche and others from Oxford Press, Norton, McGraw-Hill and American Psychiatric Publishing outside of the submitted work.

## Introduction

Since its inception in the 1980s, the internet has become a global phenomenon.^1^^–^^3^ Some adolescents and adults develop a problem controlling their use of the internet, leading to marked functional impairment (for example lower quality of life, worse scholastic outcomes and occupational difficulties).^4^ Historically, the term ‘internet addiction disorder’ started appearing in the mid-nineties^1^^–^^3^ to describe a maladaptive pattern of use of online resources that shared the characteristics of an addictive or compulsive disorder. Since then, the diagnostic criteria, assessment tools and conceptual formulation of internet addiction have been controversial.^5^^,^^6^ Theoretically different views on problematic use of the internet exist, as exemplified by the terms referred to, for example compulsive internet use, problematic internet use (PIU), internet addiction. DSM-5 features internet gaming disorder (IGD) in Section III, as a condition in need of further study, but does not include the more general disorder of PIU.^7^ DSM-5 highlights that IGD appears to be most common in male adolescents, aged 12–20 years.^7^

The concept of PIU was coined to avoid classification with addictions until more about the disorder was understood.^8^^,^^9^ It has been noted that a broad range of excessive online behaviours are associated with marked functional impairment as well as with profound psychiatric sequalae, including in adolescents,^10^ adults^11^ and mixed samples of both.^12^ Based on empirical evidence, we define PIU as excessive online activities likely to be associated with marked functional impairment, including compulsive online buying, gambling, cybersex, as well as excessive use of online streaming and social media that have addictive, impulsive and/or compulsive elements.^11^^,^^13^ Age may influence the presentation of PIU and its comorbidities. For example, one study found that attention-deficit hyperactivity disorder (ADHD) and social anxiety were associated with PIU in young adults; whereas generalised anxiety disorder and obsessive–compulsive disorder (OCD) were associated with PIU in older adults.^14^ Thus, PIU can occur in younger and older individuals but may present differently as a function of age. The debate is still ongoing as to whether PIU should be classified as an addictive, impulse control^5^ or obsessive–compulsive related disorder.^15^^,^^16^

## Neurobiology of problematic internet use

Understanding of the neurobiological underpinnings of a given mental disorder is vital for optimising disease models, classification and treatment approaches; as well as in understanding how it may relate to other disorders. In the case of excessive use of the internet, research in this area has the additional utility of helping to confirm or refute its validity. Currently, little is known about the neurocognitive determinants of PIU. Examining the cognitive performance of people with PIU to identify deficits (i.e. significantly worse performance compared against matched healthy controls) can provide insights into the neuropsychological mechanisms underpinning the disorder, and possible overlap with other psychiatric conditions. Conceptually, as noted above, PIU may share parallels with behavioural addiction, incorporating features such as escalating use over time, loss of control, concealing excessive use from others, failed attempts to cut back, and psychological distress when/if prevented from using the internet.^3^^,^^17^ In integrating research on PIU phenomena, the interaction of person-affect-cognition-execution model was developed by Brand and colleagues.^18^ Within this conceptual framework, reductions in executive functioning and inhibitory control contribute to engagement in online behaviours, leading to gratification and ultimately contributing to the emergence and persistence of PIU.

Despite growing numbers of published case–control studies examining cognition in this context, there is a paucity of rigorous meta-analyses from which to draw firm conclusions and examine potential moderators. In a meta-analysis restricted to IGD and one cognitive domain, a significant decrement was found for response inhibition compared with controls.^19^ Current models of PIU suggest that a broader range of cognitive failures may contribute including top–down inhibitory control, working memory and decision-making.^20^ The aim of the current study was to conduct a rigorous systematic review and meta-analysis of cognitive findings in PIU from case–control studies, including in adolescents and adults, reported in the peer-reviewed literature. We hypothesised, based on findings from individual studies and parallels between PIU and other related disorders, such as problematic gambling, that the condition would be associated with marked impairments across the above cognitive domains.

## Method

Our meta-analysis protocol followed the Meta-analysis Of Observational Studies in Epidemiology (MOOSE) guidelines^21^ and was preregistered electronically and published online on the PROSPERO International prospective register of systematic reviews (available from: http://www.crd.york.ac.uk/PROSPERO/display_record.php?ID=CRD42017080405).

### Search strategy

**Fig. 1:**
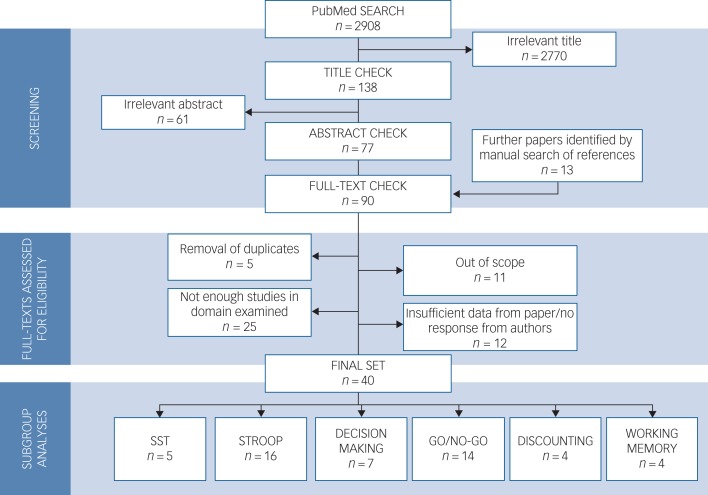
Search strategy followed for meta-analysis.

SST, stop-signal task; STROOP, Stroop attentional inhibition task; Go/No-Go, go/no-go motor inhibitory control task. Please note that some studies in the final set examined more than one domain included in the final analysis.

Our search and screening strategy is outlined in Fig. 1. The search string was determined by consensus among the coauthors. The PubMed search was conducted with the following string: [“cognitive” OR “cognition” OR “memory” OR “executive” OR “attention” OR “decision-making” OR “gambling task” OR “inhibition” OR “stroop” OR “stop-signal” OR “go no go” OR “go/no-go” OR “gng” AND “internet use” OR “internet addiction” OR “gaming addiction” OR “PIU” OR “PUI” OR “internet gaming disorder”]. The initial search yielded 2908 results. The majority of these were excluded based on reading of the title and abstract, as a result of being out-of-scope (for example papers not measuring cognition, without a suitable control group or unrelated to PIU). This yielded 138 possibly eligible papers for inclusion. We then undertook a consensus meeting involving three members of the study team and examined full texts to exclude papers that were out-of-scope; references of full-text documents were also screened for further papers within scope.

### Inclusion criteria

We included all studies that (a) were published in scholarly peer-reviewed journals between 1995 and October 2017; (b) were written in English or provided an English translation; (c) examined a cognitive domain that was also measured in at least three other studies (i.e. sufficient *n* for valid meta-analysis); (d) examined cognitive measures of participants with PIU (used in its wider meaning to include the full spectrum of ‘addictive use of the internet’, ‘problematic internet use’ and ‘internet gaming disorder’) versus healthy controls and (e) included necessary information to calculate effect sizes. Where a given paper had not reported necessary information to calculate effect sizes the study team contacted the paper's authors via email to request this information.

### Exclusion criteria

We excluded studies that (a) did not report cognitive measures; (b) used non-standard cognitive tasks (those tailored to a particular study where independent replication would not have been possible; and/or those not focusing on a recognised cognitive domain); (c) did not have a healthy comparison group; (d) lacked the required measures for meta-analysis (and such information was not provided within 4 weeks by the paper's authors); and (e) were published only in the grey literature (including conference papers, non-peer-reviewed publications, doctoral theses; as these sources are not necessarily subject to the same journal-level rigorous peer-review procedures as non-grey literature).

### Data collection and analysis

Data were extracted from the original papers or were provided by the authors of each study. Information from the included studies was recorded in an electronic spreadsheet and different types of data were extracted from each study including: (a) a geographical determinant in which the data collection occurred; (b) key demographics of the participants (age as categorised by mean age reported in the sample: children 0–12, youth 12–24, adults 24–55, older people ≥55; gender distribution in the sample as ‘male only’, ‘female only’, or ‘mixed’); (c) operationalisation of PIU including instrument used and cut-off variant; (d) reported psychiatric comorbidities in the sample; (e) effects of PIU on cognitive measures; (f) quality scores. The quality assurance control was performed independently by two psychiatrists (K.I., S.R.C.; Cohen's kappa 0.96), who then met together to arrive at a consensus. All papers in scope were assessed against the quality standard individually and received a score between 0 and 10 (for quality scoring details see supplementary material ‘Quality assurance’ and Table 1 available at https://doi.org/10.1192/bjp.2019.3).

The full list and references of studies that entered the meta-analysis are reported in supplementary Table 2. Data were analysed using statistical software R version 3.4.2. Meta-analysis was performed using packages of ‘robumeta’ and ‘metafor’.^22^ To provide a more generalisable model estimate, a random-effects model was used in all cases. The R code used for this analysis is shared in the supplement (see supplementary material ‘R code’), to support reproducible research. To compare PIU and control groups in terms of quantitative measures of cognitive performance we used mean scores and s.d. to calculate standardised mean difference measures, which were used to produce random-effects models for each different cognitive domain under investigation. Statistical significance was defined as *P* < 0.05 two-tailed throughout, and standard effect sizes were also reported. Moderator analysis was conducted to examine potential effects of the following on the results: age, gender (i.e. ‘males only’ versus ‘mixed’), presence of comorbidities (i.e. psychiatric comorbidities in the sample versus not), quality of study, whether or not online gaming was the predominant type of online activity (IGD versus PIU) and geographical area of reported study. Publication bias was assessed using regression tests for funnel plot asymmetry^23^ and, where appropriate, the trim and fill method.^24^ Heterogeneity was quantified using tau-squared and *Q*-tests. For more information about the cognitive tests included in the meta-analysis see supplementary material ‘Description of cognitive domains and key outcome measures’.

## Results

The number of data studies and total pooled sample sizes used in the meta-analysis are summarised in Table 1. Sufficient suitable data were found for meta-analysis of the following cognitive domains (tasks): motor inhibition (go/no-go), pre-potent motor inhibition (stop-signal), decision-making (Cambridge Gambling Task, Iowa Gambling Task, game of dice and Balloon Analogue Risk), working memory (digit span, spatial working memory) and discounting. The mean quality scores for the included studies, expressed as percentage of maximum, was: 68% (s.d. = 21%, range 2–9) (see supplementary Table 1 for full details). Effects of scores in moderation analysis are reported later. Most studies (approximately 80%) screened for affective disorders and substance misuse using validated instruments, whereas relatively few (<10%) screened for impulse-control disorders and gambling disorder. Another limitation of the extant data was that most studies were conducted in relatively young adults hence the association between PIU and cognition in older age groups could not be addressed.

**Table 1:** Total pooled sample sizes and model estimate measures for different cognitive domains

Domain	Studies, *n*	PIU, total *n*	Control, total *n*	Model estimate (s.e.)	*P*^a^
Attentional inhibition (Stroop)	16	362	361	0.53 (0.175)	<0.01
Motor inhibitory control (GNG)	14	330	333	0.51 (0.167)	<0.001
Motor inhibitory control (SST)	5	149	279	0.42 (0.12)	<0.001
Decision-making	7	188	349	0.54 (0.14)	<0.001
Working memory	4	126	254	0.40 (0.17)^b^	<0.05
Discounting	4	98	93	1.03 (0.26)	–^c^
Total	40	1248	1674	–	–

PIU, problematic internet use; GNG, go/no-go task; SST, stop-signal task. Some studies analysed more than one domain.

a. *P*-values here describe the probability of obtaining the observed model estimates under null hypothesis (no true differences between groups).

b. Adjusted model estimate after trim and fill method was applied because of publication bias.

c. Not further analysed because of publication bias and other methodological limitations (see supplementary material 'Discounting' and supplementary Tables 3 and 4.).

**Fig. 2:**
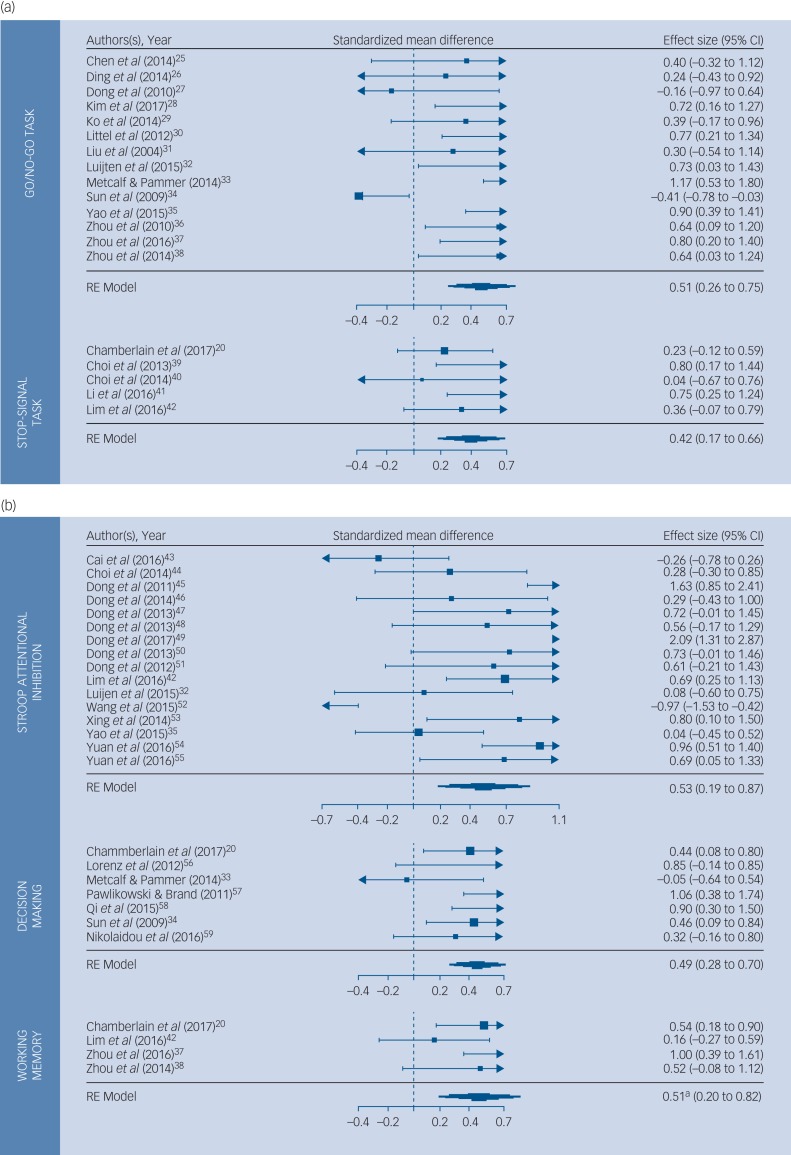
Forest plots for (a) motor inhibitory control cognitive domains; and (b) for Stroop inhibitory control, decision-making and working memory cognitive domains.

**Fig. 3:**
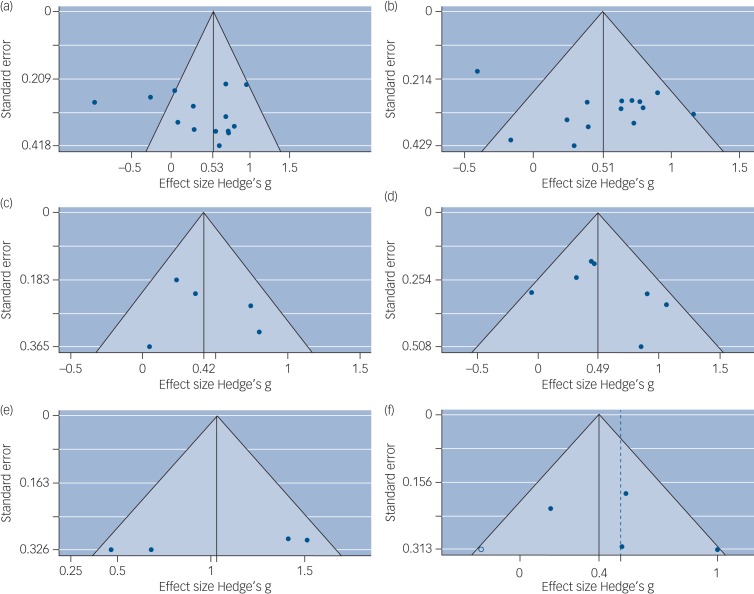
Funnel plots by cognitive domain.

Figure 2(a) shows results from the meta-analysis of motor inhibitory control domains, where it can be seen that PIU was associated with significant impairment on go/no-go and stop-signal tasks versus controls with small-medium effect sizes (Hedge's *g* = 0.51 and Hedge's *g* = 0.42, respectively, see also Fig. 3). Figure 2(b) shows meta-analytic results for the domains of attentional inhibition (colour-word Stroop), decision-making and working memory. PIU was associated with significant impairment versus controls across all three domains with small-medium effect sizes (Hedge's *g* = 0.53, Hedge's *g* = 0.49 and Hedge's *g* = 0.51, respectively). The discounting domain was excluded and not considered further due to methodological limitations (see supplementary material ‘Discounting’ and supplementary Figures 3 and 4).

Forest plots for various cognitive domains of problematic internet use participants versus controls; effect sizes are Hedge's *g*; positive values indicate people with problematic internet use performed worse than controls. ^a^Effect size for working memory domain here is reported uncorrected. RE, Random effects.

(a) Attentional inhibition (Stroop task) test for plot asymmetry: *z* = 1.77, *P* = 0.078; (b) motor inhibitory control (go/no-go task) test for plot asymmetry: *z* = 0.46, *P* = 0.64; (c) motor inhibitory control (stop-signal task) test for plot asymmetry: *z* = 0.43, *P* = 0.66; (d) decision-making test for plot asymmetry: *z* = 1.1, *P* = 0.27; (e) discounting test for plot asymmetry: *z* = –2.7670, *P* = 0.0057; (f) working memory test for plot asymmetry: *z* = 0.88, *P* = 0.37. Meta-analysis funnels plots by cognitive domain; *z-* and *P-*values reported from regression test for funnel plot asymmetry (mixed-effects meta-regression model). Evidence of publication bias identified in the domains of discounting and working memory. The trim and fill method was used although effect size changed only for working memory (as indicated by the dotted line (non-corrected effect size 0.51)).

Evidence of publication bias was observed for the working memory domain, but the finding retained statistical significance when the trim and fill approach was used (see also Fig. 3). Homogeneity metrics are presented in full in supplementary Table 5. High heterogeneity was identified in Stroop studies and low to moderate heterogeneity was found for the other examined cognitive domains.

Age, gender, presence of comorbidities, whether or not gaming was the predominant online activity and geographical area were not significant moderating factors in any of the cognitive domains examined (all *P* > 0.05 non-corrected). In some cases, analysis was not possible because of lack of comparison groups. For example, Stroop and stop-signal studies had only been performed in youth (adolescents and young adults) and Stroop studies were only performed in populations lacking comorbidities. Quality of study was a significant moderating variable in stop-signal task (*P* = 0.032) with all higher quality studies^20^^,^^25^^,^^26^ (quality mean  9/10) reporting smaller and non-statistically significant effects, and the two relatively lower quality studies^27^^,^^28^ (quality mean 7/10) reporting higher and statistically significant effects. Study quality was not a significant moderator for the other cognitive domains. More details on moderator analysis results are presented in the supplementary Table 6.

## Discussion

### Main findings

This is the first study to amass all available information from case–control studies of cognitive performance in people with PIU. We defined PIU as excessive online activities likely to be associated with marked functional impairment, including compulsive online buying, gambling, cybersex, as well as excessive use of online streaming and social media that have addictive, impulsive and/or compulsive elements. In meta-analysis, PIU was associated with significant cognitive deficits in attentional inhibition, motor inhibition (and pre-potent motor inhibition), decision-making and working memory, in line with our *a priori* hypothesis and supporting recent conceptualisations of PIU that implicate cognitive dysfunction in its pathophysiology.^17^^,^^18^

These findings were not significantly moderated by whether or not online gaming was the predominant form of online behaviour, nor by geographical site, age, gender or comorbidities. Study quality did not significantly moderate the results, except for evidence of lesser stop-signal impairment for studies that were of higher quality. These neurocognitive results support the existence of underlying frontostriatal dysfunction in PIU, and highlight the need for international collaborations using standardised measures to further elucidate its precise neurobiological underpinnings and the specificity of deficits in given domains. These findings also suggest a common neurobiological vulnerability across PIU behaviours, including gaming.

### Comparison with findings from other studies

Two previous systematic reviews examined ‘higher order’ meta-cognitive constructs that are relevant for IGD, including escapism, social identity and acceptance and beliefs about game reward,^60^^,^^61^ without providing a quantitative measure of cognition nor covering in detail neurocognitive performance. Therefore, in the wider context of existing literature, our study advances our knowledge of the neurocognitive aspects of PIU.

One previous meta-analysis of response inhibition was conducted in gaming disorder, which reported significant impairment.^19^ The current study extends beyond this prior meta-analysis by also considering the impact of study quality, and including a much larger range of available data. Problematic internet users are characterised by elevated behavioural impulsivity and compulsivity,^11^^,^^15^ which are characteristics of a wide range of psychiatric disorders, including ADHD, OCD, impulse control and substance use disorders.

### Comorbidity

The majority of studies in this meta-analysis screened for mainstream mental disorders (such as affective disorders (78%) or substance misuse (80%)) using validated instruments. However, very few indeed used appropriate screening tools to identify comorbid impulse-control disorders (for example gambling disorder, ADHD) (7.5%). As such, the current meta-analysis cannot fully assess the contribution of comorbid impulsive disorders to the observed cognitive deficits. Data elsewhere suggest that cognitive problems are more pronounced in individuals with PIU with comorbid impulse-control disorders.^62^ Nonetheless, the results of this meta-analysis demonstrate that people with PIU have measurable deficits versus controls in cognitive performance, which may have implications for day-to-day functioning, even if they partly stem from unmeasured comorbid disorders.

### Age and symptom duration

Another important aspect to consider is the effects of age and symptom duration in PIU. Although we did not find a moderating effect of participant age on the cognitive findings, most studies in this meta-analysis were conducted in relatively young participants. Excessive use of the internet can occur in older people,^14^ and this is a neglected area of research. Studies did not generally report symptom duration, so the current analysis cannot evaluate the extent to which cognitive problems may pre-date symptoms (perhaps reflecting vulnerability) as opposed to arising because of chronic engagement with internet-related activities. A longitudinal (3-months) exposure of smartphone-naive young adults to heavy smartphone use found it resulted in performance decrease in arithmetic accuracy and increase in concern for appropriateness (a measure of tendencies to conform to group conformity pressures).^63^ Although these results are preliminary, they may demonstrate the capacity of PIU to cause cognitive and behavioural changes.

### Limitations

We need to highlight that ~85% of the studies included in the meta-analysis were based in centres of predominantly Asian communities. This limits the generalisability of the results to a degree, nevertheless, there was no evidence from the moderator analysis that the geographical area of study had an impact on the observed cognitive effects. Previous work has established that PIU is a global issue,^4^ and our meta-analysis supports the notion that the neurocognitive signature of PIU is not influenced by ethnicity. This is in line with previous work, which found that the profiles of PIU were similar across two separate geographical and cultural settings (USA and South Africa).^11^ In addition, IQ measures are known to influence neurocognitive performance, which means that IQ is a parameter which needs to be controlled for in comparison studies. However, only 22.5% of studies included direct measures of IQ, and therefore, it is unclear whether differences between participants with PIU and control participants may have been caused by differences in IQ. Robust research should include such measures in the future.

Some studies were excluded due to use of non-standard cognitive domains, use of non-standard variants of common neuropsychological tasks (those not enabling replication by other groups); or insufficient numbers of other papers in the given domain to facilitate meta-analysis (a full list of those are presented in supplementary Table 7). For example, a number of studies utilised variants of the Stroop test with internet-related stimuli; pooling effects of ‘Stroop’ studies and ‘internet Stroop’ studies was not scientifically justified, because they evaluate different cognitive processes (colour-word inhibition versus attentional bias for internet-related stimuli, the latter measured via a heterogeneous spread of stimulus types and methodological approaches). By excluding these studies we do not mean to suggest that they are not extremely relevant for understanding PIU; but rather, the technique of meta-analysis is not well suited to examining non-standardised cognitive tasks, and is not suitable when few independent studies exist for a given cognitive domain. Finally, we opted for a broad operational definition of PIU; however, we recognise that further research is needed to better define and characterise PIU and its composite behaviours.

### Summary and recommendations for future studies

The current meta-analysis provides firm evidence that PIU (defined broadly and operationally) is associated with cognitive impairments in motor inhibitory control, working memory, Stroop attentional inhibition and decision-making. These findings were not moderated by age, gender, geographical location or by whether the predominant online activity was gaming or not. This analysis constitutes a vital first step towards a better understanding of PIU, supporting its existence as a biological plausible entity associated with dysfunction of frontostriatal brain circuitry, and with clinical implications for people affected by PIU. The extent to which the identified cognitive deficits were present prior to PIU, or rather stemmed from engaging in such problematic behaviours cannot be addressed within the confines of this cross-sectional data analysis. Longitudinal studies are needed to address the issue of direction of effect and causality. Based on cognitive findings in other settings, such as in the context of substance use and behavioural addiction (gambling), we theorise that some cognitive problems associated with PIU may constitute vulnerability markers; whereas others may be more associated with chronicity.^17^

This analysis also serves to highlight vital next steps needed in future papers, to further elucidate the specificity of the findings and their nature (see Appendix). This should include clarification of the role of IQ, the specific problematic behaviours involved beyond gaming, comorbid disorders that were seldom screened for (ADHD, impulse-control disorders including gambling disorder), examining a broader range of ages and other cultural settings, and employing optimised designs to maximise study quality. The review also identifies several cognitive domains that have yet to be extensively or adequately examined in PIU, such as facial processing, set-shifting, verbal recall, sustained attention, discounting, reflection-impulsivity and executive planning.

## Supplementary material

For supplementary material accompanying this paper visit https://doi.org/10.1192/bjp.2019.3. click here to view supplementary material

## Appendix

### Recommendations for future cognitive investigations of problematic internet use

(a) Salient demographic characteristics of the sample (each study group) should be described including age, gender, education levels and ethnicity.

(b) Specific problematic behaviours on the internet should be included, as this enables diagnostic specification of type of problematic internet use, for example gaming, gambling, sex, shopping, social networking, streaming media.

(c) Group differences in general intelligence should be ruled out using a suitable IQ test.

(d) When considering cognitive tests to include in a study, due consideration should be given to validation of tests in other settings and how easy it would be for other groups to attempt to replicate the findings.

(e) When describing cognitive results, inclusion of mean, standard deviations and sample size in each group is extremely valuable. For example, when using graphs, this information should also be included in a footnote in precise numerical form.

(f) Co-occurring comorbidities should be identified including mainstream mental disorders but also impulse-control disorders using suitable screening and diagnostic methods.
