# Supplementary material for: Cognitive deficits in problematic internet use: meta-analysis of 40 studies
Source: Br J Psychiatry. 2019 Nov;215(5):639–46. doi: 10.1192/bjp.2019.3 (PMC6949138; doi:10.1192/bjp.2019.3)
Supplement: Supplementary file 1 [file S0007125019000035sup001.docx]

# Quality assurance

To address the risk of bias in relation to the quality of studies included in our meta-analysis we devised a quality scoring system with which we assessed each individual study that entered the final stage of analysis. The quality assurance control was performed in a consensus meeting attended by co-authors (KI, RH, SRC, two experienced psychiatrists and one experienced research assistant). In the meeting all papers in scope were assessed against the quality standard individually and received a score between 0-10. This was then used for moderation analysis to ascertain whether the quality of studies influenced the pooled effect estimate.

In more detail, our scoring system consisted of 10 items. The items were chosen based on standard quality approaches, but also tailored to the specific parameters that the authors considered important for the specific field under study. Studies attracted one point for including each of the following 1) a screening tool for Internet addiction (including IAT, DCIA-C, YDQ etc) 2) a diagnostic interview of problematic internet use 3) a report of co-morbidities assessed using an appropriate method (e.g. MINI, DSM-IV or equivalent) including mood and anxiety disorders 4) a report of substance misuse co-morbidities assessed using an appropriate method (e.g. MINI, DSM-IV or equivalent) 5) a report of attention-deficit hyperactivity disorder assessed using an appropriate method (e.g. DIVA, ASRS or equivalent) or a measure of impulsivity (e.g. BIS) 6) a report of impulse control disorders and/or gambling assessed using an appropriate method (e.g. MIDI or equivalent) 7) an IQ measure 8) a report of level of education or equivalent 9) a report of appropriate cognitive performance measures (e.g. Stroop effect for attentional inhibition) 10) a numerical report of cognitive performance measures (e.g. mean, standard deviation or equivalent).

## TABLE S1 – Quality measures of studies

| **Paper** | **Task** | **Diagnostic Interview** | **Diagnostic screening tool** | **Mood and anxiety disorders** | **Substance misuse** | **Impulse control /gambling** | **ADHD and/or impulsivity** | **IQ measure** | **Education** | **Appropriate Cognitive performance measures** | **Data reporting** | **Total** |
| --- | --- | --- | --- | --- | --- | --- | --- | --- | --- | --- | --- | --- |
| Cai et al., Brain Imag Behav, 2016 | Stroop | 0 | 1 | 0 | 1 | 0 | 0 | 1 | 1 | 0 | 1 | 5 |
| Chamberlain et al., CNS Specs, 2017 | Decision-making | 1 | 1 | 1 | 1 | 1 | 1 | 0 | 1 | 1 | 1 | 9 |
| Chamberlain et al.,CNS Specs, 2017 | SSRT | 1 | 1 | 1 | 1 | 1 | 1 | 0 | 1 | 1 | 1 | 9 |
| Chamberlain et al.,CNS Specs, 2017 | Working memory | 1 | 1 | 1 | 1 | 1 | 1 | 0 | 1 | 1 | 1 | 9 |
| Chen et al., Psych and Clin Neuro, 2014 | GNG | 1 | 1 | 0 | 1 | 0 | 1 | 0 | 1 | 1 | 1 | 7 |
| Choi et al., Int J of Psychophys 2013 | SSRT | 0 | 1 | 1 | 1 | 0 | 1 | 1 | 1 | 0 | 1 | 7 |
| Choi et al., Journ Behav Add, 2014 | SSRT | 1 | 1 | 1 | 1 | 1 | 1 | 0 | 1 | 1 | 1 | 9 |
| Choi et al., Psych Res, 2014 | Stroop | 0 | 1 | 1 | 1 | 0 | 1 | 1 | 1 | 1 | 1 | 8 |
| Ding et al., Behav Brain Func, 2014 | GNG | 1 | 1 | 1 | 1 | 0 | 1 | 0 | 1 | 1 | 1 | 8 |
| Dong et al., Neurosci Lett, 2011 | Stroop | 0 | 1 | 0 | 0 | 0 | 0 | 0 | 1 | 0 | 1 | 3 |
| Dong et al., Addict Behav, 2014 | Stroop | 0 | 1 | 1 | 1 | 0 | 0 | 0 | 1 | 0 | 1 | 5 |
| Dong et al., Bio Psych, 2013 | Stroop | 0 | 1 | 1 | 1 | 0 | 0 | 0 | 1 | 1 | 1 | 6 |
| Dong et al., Eur Addict Res, 2013 | Stroop | 0 | 1 | 1 | 1 | 0 | 0 | 0 | 1 | 0 | 1 | 5 |
| Dong et al., Europ Psych 2017 | Stroop | 0 | 1 | 1 | 1 | 0 | 0 | 0 | 1 | 0 | 0 | 4 |
| Dong et al., Neurosci Lett, 2010 | GNG | 0 | 1 | 0 | 0 | 0 | 0 | 0 | 1 | 1 | 1 | 4 |
| Dong et al., Prog Neuro-psycho, 2013 | Stroop | 0 | 1 | 1 | 1 | 0 | 0 | 0 | 0 | 1 | 1 | 5 |
| Dong et al., Psych Res, 2012 | Stroop | 0 | 1 | 1 | 1 | 0 | 1 | 0 | 0 | 0 | 1 | 5 |
| Kim et al., Sci Re;, 2017 | GNG | 1 | 1 | 1 | 1 | 0 | 1 | 1 | 1 | 1 | 1 | 9 |
| Ko et al., Eur Arch Psych, 2014 | GNG | 1 | 1 | 1 | 1 | 0 | 1 | 0 | 1 | 0 | 1 | 7 |
| Li et al, Cyberpsychol Beh and Soc N, 2016 | Discounting | 0 | 1 | 1 | 1 | 0 | 1 | 0 | 1 | 1 | 0 | 6 |
| Li et al., Front Psychol, 2016 | SSRT | 0 | 1 | 1 | 1 | 0 | 1 | 0 | 1 | 1 | 1 | 7 |
| Lim et al., Medicine Open, 2016 | SSRT | 1 | 1 | 1 | 1 | 0 | 1 | 1 | 1 | 1 | 1 | 9 |
| Lim et al., Medicine Open, 2016 | Stroop | 1 | 1 | 1 | 1 | 0 | 1 | 1 | 1 | 1 | 0 | 8 |
| Lim et al., Medicine Open, 2016 | Working memory | 1 | 1 | 1 | 1 | 0 | 1 | 1 | 1 | 1 | 1 | 9 |
| Lin et al., Prog in Neuro-Psych, 2015 | Discounting | 0 | 1 | 0 | 0 | 0 | 0 | 0 | 0 | 1 | 0 | 2 |
| Littel et al., Addict Biol, 2012 | GNG | 0 | 1 | 1 | 0 | 0 | 1 | 0 | 1 | 1 | 1 | 6 |
| Liu et al., Kaohs Journ Med Sci, 2014 | GNG | 1 | 1 | 1 | 1 | 0 | 0 | 0 | 1 | 0 | 1 | 6 |
| Lorenz et al., Addiction Biol, 2012 | Decision-making | 0 | 1 | 1 | 1 | 0 | 1 | 0 | 1 | 1 | 1 | 7 |
| Luijten et al., Psych Res: Neuroim 2015 | Stroop | 0 | 1 | 1 | 1 | 0 | 1 | 0 | 1 | 0 | 1 | 6 |
| Luijten et al., Psych Res: Neuroim 2015 | GNG | 0 | 1 | 1 | 1 | 0 | 1 | 0 | 1 | 1 | 1 | 7 |
| Metcalf et al., Cyberpsychol Beh and Soc N, 2014 | GNG | 0 | 1 | 0 | 0 | 0 | 1 | 0 | 0 | 0 | 1 | 3 |
| Metcalf et al., Cyberpsychol Beh and Soc N, 2014 | Decision-making | 0 | 1 | 0 | 0 | 0 | 1 | 0 | 0 | 1 | 1 | 4 |
| Nikolaidou et al., J Behav Add 2016 | Decision-making | 0 | 1 | 1 | 1 | 0 | 0 | 0 | 1 | 1 | 0 | 5 |
| Pawlikowski & Brand, Psych Res, 2011 | Decision-making | 0 | 1 | 1 | 1 | 1 | 0 | 1 | 1 | 1 | 1 | 8 |
| Qi et al., Front in Behav Neuro, 2015 | Decision-making | 0 | 1 | 0 | 1 | 0 | 1 | 1 | 1 | 1 | 1 | 7 |
| Sun et al., CNS Spectr, 2009 | Decision-making | 0 | 1 | 0 | 0 | 0 | 0 | 0 | 1 | 1 | 0 | 3 |
| Sun et al., CNS Spectr, 2009 | GNG | 0 | 1 | 0 | 0 | 0 | 0 | 0 | 1 | 1 | 0 | 3 |
| Wang et al., Eur Arch Psychiatry Clin Neurosci, 2017 | Discounting | 0 | 1 | 1 | 1 | 0 | 1 | 0 | 1 | 1 | 0 | 6 |
| Wang et al., European Psych, 2016 | Discounting | 0 | 1 | 1 | 1 | 0 | 1 | 0 | 1 | 1 | 0 | 6 |
| Wang et al., Front in Behav Neuro, 2015 | Stroop | 0 | 1 | 0 | 0 | 0 | 0 | 0 | 1 | 0 | 1 | 3 |
| Xing et al., Brain Res, 2014 | Stroop | 0 | 1 | 1 | 1 | 0 | 1 | 1 | 1 | 1 | 1 | 8 |
| Yao et al., Psych Res, 2015 | GNG | 0 | 1 | 1 | 1 | 0 | 0 | 0 | 1 | 1 | 1 | 6 |
| Yao et al., Psych Res, 2015 | Stroop | 0 | 1 | 1 | 1 | 0 | 0 | 0 | 1 | 1 | 1 | 6 |
| Yuan et al., Addiction Biol, 2016 | Stroop | 0 | 1 | 1 | 1 | 0 | 1 | 1 | 1 | 0 | 1 | 7 |
| Yuan et al., Brain Struct Funct, 2016 | Stroop | 0 | 1 | 1 | 1 | 0 | 1 | 0 | 1 | 1 | 1 | 7 |
| Zhou et al, Act Neuropsychiatrica, 2010 | GNG | 0 | 1 | 1 | 1 | 0 | 1 | 0 | 1 | 0 | 1 | 6 |
| Zhou et al., Acta Neuropsychiatrica, 2016 | Working memory | 0 | 1 | 1 | 1 | 0 | 1 | 0 | 1 | 1 | 1 | 7 |
| Zhou et al., Acta Neuropsychiatrica, 2016 | GNG | 0 | 1 | 1 | 1 | 0 | 1 | 0 | 1 | 1 | 1 | 7 |
| Zhou et el., Front Behav Neurosc 2014 | Working memory | 0 | 1 | 1 | 1 | 0 | 1 | 0 | 1 | 1 | 1 | 7 |
| Zhou et el., Front Behav Neurosc 2014 | GNG | 0 | 1 | 1 | 1 | 0 | 1 | 0 | 1 | 1 | 1 | 7 |

# TABLE S2 - Full list of studies included in the meta-analysis with demographic and problematic internet use characteristics

| **ID** | **Study** | **Age** | **Gender Mix** | **Area** | **Scale** | **PIU Definition** | **Reported Comorbidities** | **Cognitive Task** | **PIU vs IGD** |
| --- | --- | --- | --- | --- | --- | --- | --- | --- | --- |
| **1** | Cai et al., Brain Imag Behav, 2016 | Youth | Mixed | China | IAT (20 items) | PIU > 50 | Healthy sample | Stroop | IGD |
| **2** | Chamberlain et al., CNS Specs, 2017 | Youth | Mixed | USA | IADQ | PIU ≥ 5 items | Mixed sample (including a % of axis-I comorbidities) | Decision-making, SSRT, Working memory | PIU |
| **3** | Chen et al., Psych and Clin Neuro, 2014 | Adults | Male only | Taiwan | DCIA | PIU ≥ 6/9 criteria A items | Healthy sample | GNG | IGD |
| **4** | Choi et al., Int J of Psychophys 2013 | Youth | Mixed | South Korea | IAT (20 items) | PIU ≥ 70 IAT score | Healthy sample | SSRT | IGD |
| **5** | Choi et al., Journ Behav Add, 2014 | Y/A* | Male only | South Korea | IAT (20 items) | PIU ≥ 70 IAT score | Healthy sample | SSRT | IGD |
| **6** | Choi et al., Psych Res, 2014 | Youth | Mixed | South Korea | IAT (20 items) | PIU ≥ 80 IAT score | Healthy sample | Stroop | PIU |
| **7** | Ding et al., Behav Brain Func, 2014 | Youth | Mixed | China | modified YDQ (8 items) | PIU ≥ 6 items | Healthy sample | GNG | PIU |
| **8** | Dong & Zhao, Neurosci Lett, 2011 | Youth | Male only | China | IAT (8 items) | PIU > 5 items | Healthy sample | Stroop | PIU |
| **9** | Dong et al., Addict Behav, 2014 | Youth | Male only | China | IAT (20 items) | PIU ≥ 80 IAT score | Healthy sample | Stroop | IGD |
| **10** | Dong et al., Bio Psych, 2013 | Youth | Male only | China | IAT (20 items) | PIU ≥ 80 IAT score | Healthy sample | Stroop | PIU |
| **11** | Dong et al., Eur Addict Res, 2013 | Youth | Male only | China | IAT (20 items) | PIU ≥ 80 IAT score | Healthy sample | Stroop | IGD |
| **12** | Dong et al., Europ Psych 2017 | Youth | Male only | China | IAT (20 items) | PIU ≥ 50 | Healthy sample | Stroop | IGD |
| **13** | Dong et al., Neurosci Lett, 2010 | Youth | Male only | China | IAT (8 items) | PIU ≥ 7 items | Healthy sample | GNG | PIU |
| **14** | Dong et al., Prog Neuro-psycho, 2013 | Youth | Male only | China | IAT (20 items) | PIU ≥ 80 IAT score | Healthy sample | Stroop | IGD |
| **15** | Dong et al., Psych Res, 2012 | Youth | Male only | China | IAT (20 items) | PIU ≥ 80 IAT score | Healthy sample | Stroop | IGD |
| **16** | Kim et al., Sci Re;, 2017 | Adult | Mixed | South Korea | Clinical Interview | Diagnostic interview | Healthy sample | GNG | IGD |
| **17** | Ko et al., Eur Arch Psych, 2014 | Adult | Male only | Taiwan | DCIA-C (Ko et al. 2009) | Diagnostic interview | Mixed sample (including a % of axis-I comorbidities) | GNG | IGD |
| **18** | Li et al, Cyberpsychol Beh and Soc N, 2016 | Youth | Mixed | China | YDQ (8 items) | PIU ≥ 6 items | Healthy sample | Discounting | PIU |
| **19** | Li et al., Front Psychol, 2016 | Youth | Mixed | China | IAT (8 items) | PIU ≥ 5 items | Healthy sample | SSRT | PIU |
| **20** | Lim et al., Medicine Open, 2016 | Youth | Male only | South Korea | DSM-5 + IAT (20 items) | PIU ≥ 70 | Healthy sample | SSRT Stroop, WM | IGD |
| **21** | Lin et al., Prog in Neuro-Psych, 2015 | Youth | Male only | China | IAT (20 items) | PIU ≥ 50 | Mixed sample (including a % of axis-I comorbidities) | Discounting | IGD |
| **22** | Littel et al., Addict Biol, 2012 | Youth | Mixed. Significant difference in gender. x^2^(1) = 16.91, P < 0.001 | Netherlands | VAT (14 items) (van Rooij et al. 2012) | PIU > 2.5 | Not reported | GNG | IGD |
| **23** | Liu et al., Kaohs Journ Med Sci, 2014 | Youth | Male only | Taiwan | DCIA-C (Ko et al. 2009) | Diagnostic interview | Mixed sample (including a % of axis-I comorbidities) | GNG | IGD |
| **24** | Lorenz et al., Addiction Biol, 2012 | Adults | Male only | Germany | WoWAI & CGB scores | Score-based criteria | Healthy sample | Iowa Gambling Task | IGD |
| **25** | Luijen et al., Psych Res: Neuroim, 2015 | Youth | Male only | Netherlands | VAT (14 items) (van Rooij et al. 2012) | PIU ≥ 2.5 | Healthy sample | GNG, Stroop | IGD |
| **26** | Metcalf et al., Cyberpsychol Beh and Soc N, 2014 | Youth | Male only | Australia | AEQ questionnaire (24 item) | PIU ≥ 4/7 core criteria | Not reported | GNG, IGT | IGD |
| **27** | Nikolaidou et al., J Behav Add 2016 | Youth | Mixed | UK | PIUQ(18 items) | PIUQ score ≥ 53 | Mixed sample (including a % of axis-I comorbidities) | IGT | IGD |
| **28** | Pawlikowski & Brand, Psych Res, 2011 | Youth | Mixed | Germany | IAT + IATwow (20 items) | PIU ≥ 50 | Healthy sample | Decision-making | IGD |
| **29** | Qi et al., Front in Behav Neuro, 2015 | Youth | Male only | China | YDQ (8 items) + IAT | PIU ≥ 5 items + ≥ 50 | Healthy sample | BART | IGD |
| **30** | Sun et al., CNS Spectr, 2009 | Youth | Mixed | China | IAT (8 items) | PIU ≥ 5 items | Healthy sample | IGT, GNG | PIU |
| **31** | Wang et al., Eur Arch Psychiatry Clin Neurosci, 2017 | Youth | Male only | China | IAT (20 items) | PIU ≥ 50 | Healthy sample | Discounting | IGD |
| **32** | Wang et al., European Psych, 2016 | Youth | Male only | China | IAT (20 items) | PIU >50 | Healthy sample | Discounting | IGD |
| **33** | Wang et al., Front in Behav Neuro, 2015 | Youth | Mixed | China | modified YDQ (8 items) | PIU ≥ 6 items | Healthy sample | Stroop | IGD |
| **34** | Xing et al., Brain Res, 2014 | Youth | Mixed | China | IAT (20 items) | PIU >50 | Healthy sample | Stroop | IGD |
| **35** | Yao et al., Psych Res, 2015 | Youth | Male only | China | CIAS 26 items | PIU ≥ 67 CIAS score | Healthy sample | Stroop, GNG | IGD |
| **36** | Yuan et al., Addiction Biol, 2016 | Youth | Mixed | China | DSM-5 | PIU ≥ 5 criteria | Healthy sample | Stroop | IGD |
| **37** | Yuan et al., Brain Struct Funct, 2016 | Youth | Mixed | China | modified YDQ (8 items) | Not reported | Healthy sample | Stroop | IGD |
| **38** | Zhou et al, Act Neuropsychiatrica, 2010 | Adult | Mixed | China | modified YDQ (8 items) | PIU ≥ 6 items | Healthy sample | GNG | PIU |
| **39** | Zhou et al., Acta Neuropsychiatrica, 2016 | Adult | Mixed | China | modified YDQ (8 items) | PIU ≥ 6 items | Healthy sample | GNG, Working memory | PIU |
| **40** | Zhou et el., Front Behav Neurosc 2014 | Adult | Mixed | China | modified YDQ (8 items) | PIU ≥ 6 items | Healthy sample | WM, GNG | PIU |

**Legend**: Full list of studies are presented here in **Table S1** (Cai et al., 2016; Chamberlain, Redden, Leppink, & Grant, 2017; C. Y. Chen et al., 2015; J. S. Choi et al., 2013, 2014; S.-W. Choi et al., 2014; Ding et al., 2014; G. Dong, Li, Wang, & Potenza, 2017; Guangheng Dong, Hu, & Lin, 2013; Guangheng Dong, Hu, Lin, & Lu, 2013; Guangheng Dong, Huang, & Du, 2011; Guangheng Dong, Lin, Zhou, & Lu, 2014; Guangheng Dong, Lu, Zhou, & Zhao, 2010; Guangheng Dong, Shen, Huang, & Du, 2013; Guangheng Dong, Zhou, & Zhao, 2011; Kim et al., 2017; C.-H. Ko et al., 2014; Li, Nan, et al., 2016; Li, Tian, et al., 2016; Lim et al., 2016; Lin, Zhou, Dong, & Du, 2015; Littel et al., 2012; Liu et al., 2014; Lorenz et al., 2013; Luijten, Meerkerk, Franken, van de Wetering, & Schoenmakers, 2015; Metcalf & Pammer, 2014; Nikolaidou, Fraser, & Hinvest, 2016; Pawlikowski & Brand, 2011; Qi et al., 2015; Sun et al., 2009; H. Wang et al., 2015; L. Wang et al., 2016; Y. Wang et al., 2017; Xing et al., 2014; Yao et al., 2015; Yuan et al., 2016, 2017; Z. H. Zhou, Yuan, Yao, Li, & Cheng, 2010; Z. Zhou, Zhou, & Zhu, 2016; Z. Zhou, Zhu, Li, & Wang, 2014). **AEQ**: Adolescent Expectancy Questionnaire; **CIAS:** Chen Internet Addiction Scale; **DCIA-C:** Diagnostic Criteria for Internet Addiction for College Students; **DSM:** Diagnostic and statistical Manual; **IAT:** Young’s Internet addiction test; **YDQ:** Young’s diagnostic questionnaire; **IADQ:** Young's Internet Addiction Diagnostic Questionnaire; **PIU:** Problematic internet use; **VAT:** Video game Addiction Test; **WoWAI:** World of Warcraft Addiction Inventory.

# Discounting

The probability discounting task is a paradigm to examine the decision-making features under risky circumstances. Pathological gamblers and participants with substance misuse have been shown to discount more steeply compared to controls (Madden, Petry, & Johnson, 2009; Petry, 2012). The preferred metric used in the meta-analysis was log(h), in which smaller values indicate greater risk seeking. Where this metric was not available, the metric log(k) was used, in which higher values indicated greater risk seeking.

We identified four studies assessing cognitive performance in internet addiction which were in scope, and according to our eligibility protocol we would have included the cognitive domain together with the remaining of the analysis. However, we encountered a number of problems during meta-analysing those studies. First, data (appropriate cognitive measures means and standard deviations) had to be extracted from graphs, which were unclear on which metric they represented; this adds an extra measurement error to our analysis. Second, the high homogeneity in conjunction with the similar demographic and performance data suggested some overlap of healthy control participants between studies included, which would be another statistical issue that would add uncertainty to the results. Finally, the low number of studies in conjunction with small study size led to publication bias. Moderator analysis had some difficulties because discounting studies had only been performed in Asian youth. Overall, we considered the domain of discounting still relatively unexplored and unclear whether confident conclusions can be drawn from this analysis. Therefore, the results are presented here, with the aforementioned caveats.

## Figure S3 – Forest plot of discounting studies


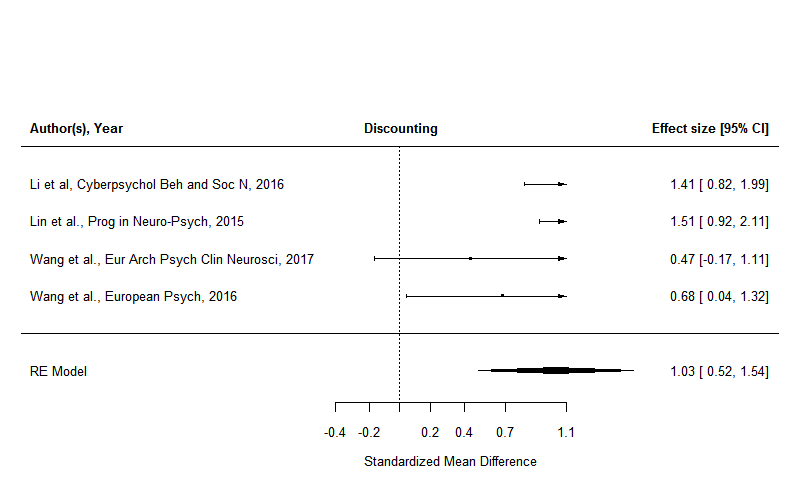


**Legend**: Forest plot showing the pooled estimate of discounting studies. Homogeneity measures were Tau^2(se): 0.172 (0.220); I^2: 63.79%; Q-test p-value: 0.040 (suggesting studies share a common effect size); Model estimate: 1.03 (0.26)

## Figure S4 – Funnel plot of discounting studies


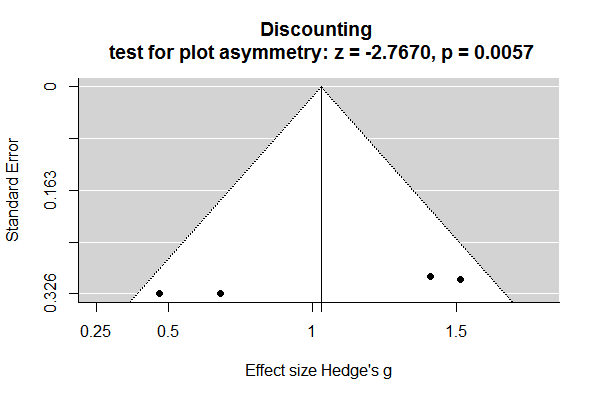


**Legend**: Meta-analysis funnels plots by cognitive domain; ‘z’ and ‘p’ values reported from Regression Test for Funnel Plot Asymmetry (mixed-effects meta-regression model). Evidence of publication bias identified in the domain of Discounting (Egger’s test p<0.01)

# TABLE S5 – Heterogeneity and model estimate measures for different cognitive domains

| **Domain** | **N studies** | **tau^2 (se)** | **I^2** | **H^2** | **Q-test**  **(p value)** † | **Model Estimate (se)** | **sig.** |
| --- | --- | --- | --- | --- | --- | --- | --- |
| **Attentional Inhibition (Stroop)** | 16 | 0.381 (0.178) | 79.72% | 4.93 | < .0001 | 0.53 (0.175) | ** |
| **Motor inhibitory control (GNG)** | 14 | 0.124 (0.086) | 57.77% | 2.37 | < .001 | 0.51 (0.167) | *** |
| **Motor inhibitory control (SST)** | 5 | 0.015 (0.054) | 19.18% | 1.24 | 0.26 | 0.42 (0.12) | *** |
| **Decision-Making** | 7 | 0.012 (0.044) | 14.96% | 1.18 | 0.17 | 0.49 (0.11) | ** |
| **Working Memory** | 4 | 0.039 (0.081) | 39.65% | 1.66 | 0.168 | 0.40 (0.17)†† | * |
| **Discounting** | 4 | 0.172  (0.22) | 63.79% | 2.76 | 0.041 | 1.03 (0.26) | N/A |

**Legend: tau^2:** estimated amount of total heterogeneity; **I^2:** (total heterogeneity / total variability); **H^2:** (total variability / sampling variability); **Q-test:** Test for Heterogeneity; meta-analysis was done using random-effects model using REML. **REML:** Restricted maximum-likelihood estimator

# TABLE S6 – Moderator analysis results

| **Domain** | **Age** | **Gender** | **Presence of co-morbidities** | **Diagnostic conceptualization (IGD vs PIU)** | **Geographical Area** | **Quality of studies** |
| --- | --- | --- | --- | --- | --- | --- |
| **Attentional Inhibition (Stroop)** | - | n.s. | - | n.s. | n.s. | n.s. |
| **Motor inhibitory control (GNG)** | n.s. | n.s. | n.s. | n.s. | n.s. | n.s. |
| **Motor inhibitory control (SST)** | - | n.s. | n.s. | n.s. | n.s. | **p = 0.03**  ***** |
| **Decision-Making** | n.s. | n.s. | n.s. | n.s. | n.s. | n.s. |
| **Working Memory** | n.s. | n.s. | n.s. | n.s. | n.s. | n.s. |

**Legend: Age** was not a moderating variable in Go/No-Go (p=0.15), decision-making (p=0.62), working memory (p=0.21) studies. There is a clear gender bias in the study designs where there was a significant number of ‘males only’ studies (~54%), but none of ‘females only’. **Gender** (analysed as ‘males only’ vs ‘mixed’) was not a moderating variable in any domain [Stroop (p=0.17), SST (p=0.65), Go/No-Go (p=0.81), decision-making (p=0.21), and working memory (p=0.071)]. Geographical area (analysed as binary variable of predominantly Asian versus predominantly Caucasian populated areas) was not a moderating variable in any domain [Stroop (p=0.52), SST (p=0.15), Go/No-Go (p=0.53), decision-making (p=0.88) and working memory (p=0.97)]. The **presence of comorbidities** was not a moderating variable in SST (p=0.15), Go/No-Go (p=0.81), decision-making (p=0.34) and working memory (p=0.97) studies. **Diagnostic conceptualization** was not a moderating variable in any domain [Stroop (p=0.40), Go/No-Go (p=0.06), decision-making (p=0.37), working memory (p=0.07)]. **Quality** was not a moderating variable in Stroop (p=0.87), Go/No-Go (p=0.22), decision-making (p=0.39) or working-memory (p=0.21).

# TABLE S7 - List of studies that were excluded from the meta-analysis

|  | **Study** | **Task** |
| --- | --- | --- |
| **1** | Bailey et al., Front in Psychol 2013 | IGT/ Temporal Discounting/Risk task/Probabilistic selection |
| **2** | Balconi & Finocchiaro, Acta Neuro, 2016 | GNG |
| **3** | Chen et al, Psych Res 2017 | Facial Processing |
| **4** | Choi et al., Psych Res, 2014 | SSRT |
| **5** | Decker et al., Comp in Human Behav, 2011 | Internet GNG |
| **6** | Dieter et al., Bheavior Brain Res 2017 | Emotional Stroop |
| **7** | Dong et al., Prog Neuropsychopharm Bio, 2015 | Stroop |
| **8** | Han et al., Journ Affec Dis 2016 | Set shifting |
| **9** | Jeromin et al,. J of Behav Add, 2016 (b) | Internet Stroop/Stroop |
| **10** | Jeromin et al,. J of Behav Add, 2016 | Internet Stroop |
| **11** | Ko et al., Asia-Pacific Psych, 2014 | Directed forgetting Internet |
| **12** | Lee et al., Cyberpsychol, 2015 | Stroop Match-to-Sample |
| **13** | Lim et al., Medicine Open, 2016 | Verbal fluency |
| **14** | Lim et al., Medicine Open, 2016 | Planning |
| **15** | Liu et al., Neuroimage, 2017 | Cups task |
| **16** | Li et al., Sci Rep, 2014 | GNG |
| **17** | Lorenz et al., Addiction Biol, 2012 | Dot probe |
| **18** | Metcalf et al., Cyberpsychol Beh and Soc N, 2014 | Sustained attention |
| **19** | Nie et al., Compr Psych, 2017 | Verbal Fluency |
| **20** | Nie et al., Psych Res, 2016 | SSRT |
| **21** | Park et al, Medicine 2017 | Auditory odd ball |
| **22** | Park et at., Transl Psych 2016 | Auditory odd ball |
| **23** | Peng et al, Front in Behav Neuro, 2017 | Facial Processing |
| **24** | Seok et al, ANZJP, 2015 | Decision-making |
| **25** | Sun et al., Cyberpsychol Beh, 2008 | Multiple Object Tracking Task |
| **26** | Vila et al., Adicciones, 2017 | Verbal fluency/ Sustained attention/ Verbal recall |
| **27** | Wang et el,. Front Behav Neursc 2015 | Stroop |
| **28** | Wang et al,. CNS Specs, 2017 | Stroop / Internet Stroop |
| **29** | Wang et al,. Addiction Biol 2016 | Discounting |
| **30** | Yao et al., PLOS ONE, 2015 | Cups task |
| **31** | Yao et al., Psych Res, 2015 | Cups task |
| **32** | Yau et al., Add Behav , 2015 | BART |
| **33** | Yen et al., Psych Res, 2011 | Implicit association Task |
| **34** | Zhang et al., Front in Psychol, 2016 | Internet Stroop |
| **35** | Zhou et al., PLOS ONE, 2012 | Internet Game-shifting Task |
| **36** | Zhou et al., Acta Neuropsychiatrica, 2016 | Set shifting |
| **37** | Zhou et al., Front Beh Neurosc 2013 | Flanker Task |

**Legend**: Studies excluded from the meta-analysis due to 1) Less than 4 studies per cognitive domain assessed; 2) Data (i.e. mean and standard deviation) not provided in original publication, nor by direct contact with authors 3) Lack of control group; (Balconi & Finocchiaro, 2016; Z. Chen, Poon, & Cheng, 2017; J. S. Choi et al., 2014; Dieter et al., 2017; Han, Kim, Bae, Renshaw, & Anderson, 2016; Jeromin, Nyenhuis, & Barke, 2016; Kim et al., 2017; C. H. Ko et al., 2015; Lee et al., 2015; Lin et al., 2015; Lorenz et al., 2013; Marín Vila, Carballo Crespo, & Coloma Carmona, 2017; Nie, Zhang, Chen, & Li, 2016; Nie, Zhang, & Liu, 2017; Seok, Lee, Sohn, & Sohn, 2015; Lingxiao Wang et al., 2017; Y. Wang et al., 2016; Yau, Potenza, Mayes, & Crowley, 2015; Zhang et al., 2016; Z. Zhou, Li, & Zhu, 2013; Z. Zhou et al., 2016, 2014)

## R code

# Use the following the command to load the data. Only sample of code is presented here, repeated sequences of code are omitted.

df <- read.csv("~/…/df_piu_cog_meta.csv", head = TRUE, stringsAsFactors = FALSE); df <- data.table(df)

#df <- mutate(df, study_id = 1:92) # This adds a study id column

df <- df %>% select(ID, paper, Age:Geographical, Co.morbidities:control.N) # This brings the study id column to the front

df_Stroop <- df %>% filter(Task == "Stroop") %>%

dplyr::select(ID, paper, piu.mean:control.N) %>%

filter(complete.cases(.))

df_Stroop <- escalc(measure="SMD", m1i=piu.mean, m2i=control.mean, sd1i=piu.SD, sd2i=control.SD, n1i=piu.N, n2i=control.N, data=df_Stroop, slab=paper)

# Now you're ready to perform the meta-analysis using a random-effects model. The following commands will print out the data and also calculates and print the confidence interval for the amount of heterogeneity (I^2).

res_Stroop <- rma(yi, vi, data=df_Stroop) ; res_Stroop #results

par(font=1); #forest plot

forest(res_Stroop, xlim=c(-2.5,2.5),

at=transf.rtoz(c(-0.6,-.4,-.2,0,.2,.4,.6, .8)), digits=c(2,1), cex=.8)

par(font=2, cex=1)

text(-2.5, 17.5, "Author(s), Year", pos=4, cex=.8)

text(-0.8, 17.5, "Attentional Inhibition (Stroop Task)", pos=4, cex=.8)

text( 2.5, 17.5, "Effect size [95% CI]", pos=2, cex=.8)

### funnel plot

par(font=1, cex=1)

funnel(res_Stroop, xlab = "Effect size Hedge's g", at= c(-0.5, 0, as.numeric(res_Stroop$beta), 1, 1.5),

main= "Attentional Inhibition (Stroop Task) \n test for plot asymmetry: z = 1.77, p = 0.078")

res.tf <- trimfill(res_Stroop)

res.tf

regtest(res_Stroop)

ranktest(res_Stroop)

#repeat code for different domains

#moderator analysis

df <- df %>% select(ID, paper, Age:Geographical, Co.morbidities:control.N) # This brings the study id column to the front

df$Age <- as.factor(df$Age); df$Co.morbidities <- as.factor(df$Co.morbidities)

dfm_Stroop <- df %>% filter(Task == "Stroop") %>%

dplyr::select(ID:control.N) %>%

filter(complete.cases(.))

res.modage <- rma(yi, vi, mods = ~ Age, data=dfm_Stroop) ;res.modage

res.modgender <- rma(yi, vi, mods = ~ Gender, data=dfm_Stroop) ;res.modgender

res.modgeo <- rma(yi, vi, mods = ~ Geographical, data=dfm_Stroop) ;res.modgeo

res.modmorb <- rma(yi, vi, mods = ~ Co.morbidities, data=dfm_Stroop) ;res.modmorb

## References

Balconi, M., & Finocchiaro, R. (2016). Deficit in rewarding mechanisms and prefrontal left/right cortical effect in vulnerability for internet addiction. *Acta Neuropsychiatrica*, *28*(05), 272–285. http://doi.org/10.1017/neu.2016.9

Barch, D. M., Carter, C. S., & Cohen, J. D. (2004). Factors Influencing Stroop Performance in Schizophrenia. *Neuropsychology*, *18*(3), 477–484. http://doi.org/10.1037/0894-4105.18.3.477

Bechara, A., Damasio, A. R., Damasio, H., & Anderson, S. W. (1994). Insensitivity to future consequences following damage to human prefrontal cortex. *Cognition*, *50*(1–3), 7–15. Retrieved from http://www.ncbi.nlm.nih.gov/pubmed/8039375

Brand, M., Recknor, E. C., Grabenhorst, F., & Bechara, A. (2007). Decisions under ambiguity and decisions under risk: Correlations with executive functions and comparisons of two different gambling tasks with implicit and explicit rules. *Journal of Clinical and Experimental Neuropsychology*, *29*(1), 86–99. http://doi.org/10.1080/13803390500507196

Cai, C., Yuan, K., Yin, J., Feng, D., Bi, Y., Li, Y., … Tian, J. (2016). Striatum morphometry is associated with cognitive control deficits and symptom severity in internet gaming disorder. *Brain Imaging and Behavior*, *10*(1), 12–20. http://doi.org/10.1007/s11682-015-9358-8

Chamberlain, S. R., Redden, S. A., Leppink, E., & Grant, J. E. (2017). Problematic internet use in gamblers: impact on clinical and cognitive measures. *CNS Spectrums*, 1–9. http://doi.org/10.1017/S1092852917000037

Chamberlain, S. R., & Sahakian, B. J. (2007). The neuropsychiatry of impulsivity. *Current Opinion in Psychiatry*, *20*(3), 255–261. http://doi.org/10.1097/YCO.0b013e3280ba4989

Chen, C. Y., Huang, M. F., Yen, J. Y., Chen, C. S., Liu, G. C., Yen, C. F., & Ko, C. H. (2015). Brain correlates of response inhibition in Internet gaming disorder. *Psychiatry and Clinical Neurosciences*, *69*(4), 201–209. http://doi.org/10.1111/pcn.12224

Chen, Z., Poon, K. T., & Cheng, C. (2017). Deficits in recognizing disgust facial expressions and Internet addiction: Perceived stress as a mediator. *Psychiatry Research*, *254*, 211–217. http://doi.org/10.1016/j.psychres.2017.04.057

Choi, J. S., Park, S. M., Lee, J., Hwang, J. Y., Jung, H. Y., Choi, S. W., … Lee, J. Y. (2013). Resting-state beta and gamma activity in internet addiction. *International Journal of Psychophysiology*, *89*(3), 328–333. http://doi.org/10.1016/j.ijpsycho.2013.06.007

Choi, J. S., Park, S. M., Roh, M. S., Lee, J. Y., Park, C. Bin, Hwang, J. Y., … Jung, H. Y. (2014). Dysfunctional inhibitory control and impulsivity in Internet addiction. *Psychiatry Research*, *215*(2), 424–428. http://doi.org/10.1016/j.psychres.2013.12.001

Choi, S.-W., Kim, H., Kim, G.-Y., Jeon, Y., Park, S., Lee, J.-Y., … Kim, D.-J. (2014). Similarities and differences among Internet gaming disorder, gambling disorder and alcohol use disorder: A focus on impulsivity and compulsivity. *Journal of Behavioral Addictions*, *3*(4), 246–253. http://doi.org/10.1556/JBA.3.2014.4.6

Dieter, J., Hoffmann, S., Mier, D., Reinhard, I., Beutel, M., Vollstädt-Klein, S., … Leménager, T. (2017). The role of emotional inhibitory control in specific internet addiction – an fMRI study. *Behavioural Brain Research*, *324*, 1–14. http://doi.org/10.1016/j.bbr.2017.01.046

Ding, W., Sun, J., Sun, Y., Chen, X., Zhou, Y., Zhuang, Z., … Du, Y. (2014). Trait impulsivity and impaired prefrontal impulse inhibition function in adolescents with internet gaming addiction revealed by a Go/No-Go fMRI study. *Behavioral and Brain Functions*, *10*(1), 20. http://doi.org/10.1186/1744-9081-10-20

Dong, G., Hu, Y., & Lin, X. (2013). Reward/punishment sensitivities among internet addicts: Implications for their addictive behaviors. *Progress in Neuro-Psychopharmacology and Biological Psychiatry*, *46*, 139–145. http://doi.org/10.1016/j.pnpbp.2013.07.007

Dong, G., Hu, Y., Lin, X., & Lu, Q. (2013). What makes Internet addicts continue playing online even when faced by severe negative consequences? Possible explanations from an fMRI study. *Biological Psychology*, *94*(2), 282–289. http://doi.org/10.1016/j.biopsycho.2013.07.009

Dong, G., Huang, J., & Du, X. (2011). Enhanced reward sensitivity and decreased loss sensitivity in Internet addicts: An fMRI study during a guessing task. *Journal of Psychiatric Research*, *45*(11), 1525–1529. http://doi.org/10.1016/j.jpsychires.2011.06.017

Dong, G., Li, H., Wang, L., & Potenza, M. N. (2017). Cognitive control and reward/loss processing in Internet gaming disorder: Results from a comparison with recreational Internet game-users. *European Psychiatry*, *44*, 30–38. http://doi.org/10.1016/j.eurpsy.2017.03.004

Dong, G., Lin, X., Zhou, H., & Lu, Q. (2014). Cognitive flexibility in internet addicts: FMRI evidence from difficult-to-easy and easy-to-difficult switching situations. *Addictive Behaviors*, *39*(3), 677–683. http://doi.org/10.1016/j.addbeh.2013.11.028

Dong, G., Lu, Q., Zhou, H., & Zhao, X. (2010). Impulse inhibition in people with Internet addiction disorder: Electrophysiological evidence from a Go/NoGo study. *Neuroscience Letters*, *485*(2), 138–142. http://doi.org/10.1016/j.neulet.2010.09.002

Dong, G., Shen, Y., Huang, J., & Du, X. (2013). Impaired error-monitoring function in people with internet addiction disorder: An event-related fMRI study. *European Addiction Research*, *19*(5), 269–275. http://doi.org/10.1159/000346783

Dong, G., Zhou, H., & Zhao, X. (2011). Male Internet addicts show impaired executive control ability: Evidence from a color-word Stroop task. *Neuroscience Letters*, *499*(2), 114–118. http://doi.org/10.1016/j.neulet.2011.05.047

Fillmore, M. T. (2003). Drug Abuse as a Problem of Impaired Control: Current Approaches and Findings. *Behavioral and Cognitive Neuroscience Reviews*, *2*(3), 179–197. http://doi.org/10.1177/1534582303257007

Han, D. H., Kim, S. M., Bae, S., Renshaw, P. F., & Anderson, J. S. (2016). A failure of suppression within the default mode network in depressed adolescents with compulsive internet game play. *Journal of Affective Disorders*, *194*, 57–64. http://doi.org/10.1016/j.jad.2016.01.013

Jeromin, F., Nyenhuis, N., & Barke, A. (2016). Attentional bias in excessive Internet gamers: Experimental investigations using an addiction Stroop and a visual probe. *Journal of Behavioral Addictions*, *5*(1), 32–40. http://doi.org/10.1556/2006.5.2016.012

Kim, M., Lee, T. H., Choi, J.-S., Kwak, Y. Bin, Hwang, W. J., Kim, T., … Kwon, J. S. (2017). Neurophysiological correlates of altered response inhibition in internet gaming disorder and obsessive-compulsive disorder: Perspectives from impulsivity and compulsivity. *Scientific Reports*, *7*(September 2016), 41742. http://doi.org/10.1038/srep41742

Kirby, K. N., & Finch, J. C. (2010). The hierarchical structure of self-reported impulsivity. *Personality and Individual Differences*, *48*(6), 704–713. http://doi.org/10.1016/j.paid.2010.01.019

Ko, C.-H., Hsieh, T.-J., Chen, C.-Y., Yen, C.-F., Chen, C.-S., Yen, J.-Y., … Liu, G.-C. (2014). Altered brain activation during response inhibition and error processing in subjects with Internet gaming disorder: a functional magnetic imaging study. *European Archives of Psychiatry and Clinical Neuroscience*, *264*(8), 661–672. http://doi.org/10.1007/s00406-013-0483-3

Ko, C. H., Wang, P. W., Liu, T. L., Yen, C. F., Chen, C. S., & Yen, J. Y. (2015). The inhibition of proactive interference among adults with Internet gaming disorder. *Asia-Pacific Psychiatry*, *7*(2), 143–152. http://doi.org/10.1111/appy.12134

Lee, J., Lee, S., Chun, J. W., Cho, H., Kim, D., & Jung, Y.-C. (2015). Compromised Prefrontal Cognitive Control Over Emotional Interference in Adolescents with Internet Gaming Disorder. *Cyberpsychology, Behavior, and Social Networking*, *18*(11), 661–668. http://doi.org/10.1089/cyber.2015.0231

Lejuez, C. W., Read, J. P., Kahler, C. W., Richards, J. B., Ramsey, S. E., Stuart, G. L., … Brown, R. A. (2002). Evaluation of a behavioral measure of risk taking: The Balloon Analogue Risk Task (BART). *Journal of Experimental Psychology: Applied*, *8*(2), 75–84. http://doi.org/10.1037/1076-898X.8.2.75

Li, Q., Nan, W., Taxer, J., Dai, W., Zheng, Y., & Liu, X. (2016). Problematic Internet users show impaired inhibitory control and risk taking with losses: Evidence from stop signal and mixed gambles tasks. *Frontiers in Psychology*, *7*(MAR), 1–9. http://doi.org/10.3389/fpsyg.2016.00370

Li, Q., Tian, M., Taxer, J., Zheng, Y., Wu, H., Sun, S., & Liu, X. (2016). Problematic Internet Users’ Discounting Behaviors Reflect an Inability to Delay Gratification, Not Risk Taking. *Cyberpsychology, Behavior, and Social Networking*, *19*(3), 172–178. http://doi.org/10.1089/cyber.2015.0295

Lim, J.-A., Lee, J.-Y., Jung, H. Y., Sohn, B. K., Choi, S.-W., Kim, Y. J., … Choi, J.-S. (2016). Changes of quality of life and cognitive function in individuals with Internet gaming disorder. *Medicine*, *95*(50), e5695. http://doi.org/10.1097/MD.0000000000005695

Lin, X., Zhou, H., Dong, G., & Du, X. (2015). Impaired risk evaluation in people with internet gaming disorder: fMRI evidence from a probability discounting task. *Progress in Neuro-Psychopharmacology and Biological Psychiatry*, *56*, 142–148. http://doi.org/10.1016/j.pnpbp.2014.08.016

Littel, M., Van Den Berg, I., Luijten, M., Van Rooij, A. J., Keemink, L., & Franken, I. H. A. (2012). Error processing and response inhibition in excessive computer game players: An event-related potential study. *Addiction Biology*, *17*(5), 934–947. http://doi.org/10.1111/j.1369-1600.2012.00467.x

Liu, G. C., Yen, J. Y., Chen, C. Y., Yen, C. F., Chen, C. S., Lin, W. C., & Ko, C. H. (2014). Brain activation for response inhibition under gaming cue distraction in internet gaming disorder. *Kaohsiung Journal of Medical Sciences*, *30*(1), 43–51. http://doi.org/10.1016/j.kjms.2013.08.005

Lorenz, R. C., Krüger, J.-K., Neumann, B., Schott, B. H., Kaufmann, C., Heinz, A., & Wüstenberg, T. (2013). Cue reactivity and its inhibition in pathological computer game players. *Addiction Biology*, *18*(1), 134–146. http://doi.org/10.1111/j.1369-1600.2012.00491.x

Luijten, M., Meerkerk, G.-J., Franken, I. H. A., van de Wetering, B. J. M., & Schoenmakers, T. M. (2015). An fMRI study of cognitive control in problem gamers. *Psychiatry Research: Neuroimaging*, *231*(3), 262–268. http://doi.org/10.1016/j.pscychresns.2015.01.004

MacLeod, C. M. (2015). The Stroop Effect. *Encyclopedia of Color Science and Technology*, 1–6. http://doi.org/10.1007/978-3-642-27851-8_67-1

Madden, G. J., Petry, N. M., & Johnson, P. S. (2009). Pathological gamblers discount probabilistic rewards less steeply than matched controls. *Experimental and Clinical Psychopharmacology*, *17*(5), 283–290. http://doi.org/10.1037/a0016806

Marín Vila, M., Carballo Crespo, J. L., & Coloma Carmona, A. (2017). Academic outcomes and cognitive performance in problematic Internet users. *Adicciones*, *0*(0), 844. http://doi.org/10.20882/adicciones.844

Metcalf, O., & Pammer, K. (2014). Impulsivity and related neuropsychological features in regular and addictive first person shooter gaming. *Cyberpsychology, Behavior and Social Networking*, *17*(3), 147–152. http://doi.org/10.1089/cyber.2013.0024

Nie, J., Zhang, W., Chen, J., & Li, W. (2016). Impaired inhibition and working memory in response to internet-related words among adolescents with internet addiction: A comparison with attention-deficit/hyperactivity disorder. *Psychiatry Research*, *236*, 28–34. http://doi.org/10.1016/j.psychres.2016.01.004

Nie, J., Zhang, W., & Liu, Y. (2017). Exploring depression, self-esteem and verbal fluency with different degrees of internet addiction among Chinese college students. *Comprehensive Psychiatry*, *72*, 114–120. http://doi.org/10.1016/j.comppsych.2016.10.006

Nikolaidou, M., Fraser, D. S., & Hinvest, N. (2016). Physiological markers of biased decision-making in problematic Internet users. *Journal of Behavioral Addictions*, *5*(3), 510–517. http://doi.org/10.1556/2006.5.2016.052

Owen, A. M., Herrod, N. J., Menon, D. K., Clark, J. C., Downey, S. P., Carpenter, T. A., … Pickard, J. D. (1999). Redefining the functional organization of working memory processes within human lateral prefrontal cortex. *The European Journal of Neuroscience*, *11*(2), 567–74. Retrieved from http://www.ncbi.nlm.nih.gov/pubmed/10051756

Pawlikowski, M., & Brand, M. (2011). Excessive Internet gaming and decision making: Do excessive World of Warcraft players have problems in decision making under risky conditions? *Psychiatry Research*, *188*(3), 428–433. http://doi.org/10.1016/j.psychres.2011.05.017

Petry, N. M. (2012). Discounting of probabilistic rewards is associated with gambling abstinence in treatment-seeking pathological gamblers. *Journal of Abnormal Psychology*, *121*(1), 151–159. http://doi.org/10.1037/a0024782

Qi, X., Du, X., Yang, Y., Du, G., Gao, P., Zhang, Y., … Zhang, Q. (2015). Decreased modulation by the risk level on the brain activation during decision making in adolescents with internet gaming disorder. *Frontiers in Behavioral Neuroscience*, *9*(November), 1–8. http://doi.org/10.3389/fnbeh.2015.00296

Seok, J.-W., Lee, K. H., Sohn, S., & Sohn, J.-H. (2015). Neural substrates of risky decision making in individuals with Internet addiction. *Australian & New Zealand Journal of Psychiatry*, *49*(10), 923–932. http://doi.org/10.1177/0004867415598009

Simon, S. L., Domier, C. P., Sim, T., Richardson, K., Rawson, R. A., & Ling, W. (2002). Cognitive performance of current methamphetamine and cocaine abusers. *Journal of Addictive Diseases*, *21*(1), 61–74. Retrieved from http://www.ncbi.nlm.nih.gov/pubmed/11831501

Sun, D.-L., Chen, Z.-J., Ma, N., Zhang, X.-C., Fu, X.-M., & Zhang, D.-R. (2009). Decision-making and prepotent response inhibition functions in excessive internet users. *CNS Spectrums*, *14*(2), 75–81. http://doi.org/10.1017/s1092852900000225

Sweeney, J. A., Kmiec, J. A., & Kupfer, D. J. (2000). Neuropsychologic impairments in bipolar and unipolar mood disorders on the CANTAB neurocognitive battery. *Biological Psychiatry*, *48*(7), 674–84. Retrieved from http://www.ncbi.nlm.nih.gov/pubmed/11032979

Torregrossa, M. M., Quinn, J. J., & Taylor, J. R. (2008). Impulsivity, compulsivity, and habit: the role of orbitofrontal cortex revisited. *Biological Psychiatry*, *63*(3), 253–5. http://doi.org/10.1016/j.biopsych.2007.11.014

Verbruggen, F., & Logan, G. D. (2008). Response inhibition in the stop-signal paradigm. *Trends in Cognitive Sciences*, *12*(11), 418–24. http://doi.org/10.1016/j.tics.2008.07.005

Wang, H., Jin, C., Yuan, K., Shakir, T. M., Mao, C., Niu, X., … Zhang, M. (2015). The alteration of gray matter volume and cognitive control in adolescents with internet gaming disorder. *Frontiers in Behavioral Neuroscience*, *9*(March), 64. http://doi.org/10.3389/fnbeh.2015.00064

Wang, L., Wu, L., Lin, X., Zhang, Y., Zhou, H., Du, X., & Dong, G. (2016). Dysfunctional default mode network and executive control network in people with Internet gaming disorder: Independent component analysis under a probability discounting task. *European Psychiatry*, *34*, 36–42. http://doi.org/10.1016/j.eurpsy.2016.01.2424

Wang, L., Zhang, Y., Lin, X., Zhou, H., Du, X., & Dong, G. (2017). Group independent component analysis reveals alternation of right executive control network in Internet gaming disorder. *CNS Spectrums*, 1–11. http://doi.org/10.1017/S1092852917000360

Wang, Y., Wu, L., Wang, L., Zhang, Y., Du, X., & Dong, G. (2016). Impaired decision-making and impulse control in Internet gaming addicts: Evidence from the comparison with recreational Internet game users. *Addiction Biology*. http://doi.org/10.1111/adb.12458

Wang, Y., Wu, L., Zhou, H., Lin, X., Zhang, Y., Du, X., & Dong, G. (2017). Impaired executive control and reward circuit in Internet gaming addicts under a delay discounting task: independent component analysis. *European Archives of Psychiatry and Clinical Neuroscience*, *267*(3), 245–255. http://doi.org/10.1007/s00406-016-0721-6

Weafer, J., Milich, R., & Fillmore, M. T. (2011). Behavioral components of impulsivity predict alcohol consumption in adults with ADHD and healthy controls. *Drug and Alcohol Dependence*, *113*(2–3), 139–146. http://doi.org/10.1016/j.drugalcdep.2010.07.027

Winstanley, C. A., & Clark, L. (2016). Translational Models of Gambling-Related Decision-Making. *Current Topics in Behavioral Neurosciences*, *28*, 93–120. http://doi.org/10.1007/7854_2015_5014

Xing, L., Yuan, K., Bi, Y., Yin, J., Cai, C., Feng, D., … Tian, J. (2014). Reduced fiber integrity and cognitive control in adolescents with internet gaming disorder. *Brain Research*, *1586*, 109–117. http://doi.org/10.1016/j.brainres.2014.08.044

Yao, Y. W., Wang, L. J., Yip, S. W., Chen, P. R., Li, S., Xu, J., … Fang, X. Y. (2015). Impaired decision-making under risk is associated with gaming-specific inhibition deficits among college students with Internet gaming disorder. *Psychiatry Research*, *229*(1–2), 302–309. http://doi.org/10.1016/j.psychres.2015.07.004

Yau, Y. H. C., Potenza, M. N., Mayes, L. C., & Crowley, M. J. (2015). Blunted feedback processing during risk-taking in adolescents with features of problematic Internet use. *Addictive Behaviors*, *45*, 156–163. http://doi.org/10.1016/j.addbeh.2015.01.008

Yuan, K., Qin, W., Yu, D., Bi, Y., Xing, L., Jin, C., & Tian, J. (2016). Core brain networks interactions and cognitive control in internet gaming disorder individuals in late adolescence/early adulthood. *Brain Structure and Function*, *221*(3), 1427–1442. http://doi.org/10.1007/s00429-014-0982-7

Yuan, K., Yu, D., Cai, C., Feng, D., Li, Y., Bi, Y., … Tian, J. (2017). Frontostriatal circuits, resting state functional connectivity and cognitive control in internet gaming disorder. *Addiction Biology*, *22*(3), 813–822. http://doi.org/10.1111/adb.12348

Zhang, Y., Lin, X., Zhou, H., Xu, J., Du, X., & Dong, G. (2016). Brain activity toward gaming-related cues in internet gaming disorder during an addiction Stroop task. *Frontiers in Psychology*, *7*(MAY), 1–9. http://doi.org/10.3389/fpsyg.2016.00714

Zhou, Z. H., Yuan, G. Z., Yao, J. J., Li, C., & Cheng, Z. H. (2010). An event-related potential investigation of deficient inhibitory control in individuals with pathological Internet use. *Acta Neuropsychiatrica*, *22*(5), 228–236. http://doi.org/10.1111/j.1601-5215.2010.00444.x

Zhou, Z., Li, C., & Zhu, H. (2013). An Error-Related Negativity Potential Investigation of Response Monitoring Function in Individuals with Internet Addiction Disorder. *Frontiers in Behavioral Neuroscience*, *7*(September), 1–8. http://doi.org/10.3389/fnbeh.2013.00131

Zhou, Z., Zhou, H., & Zhu, H. (2016). Working memory, executive function and impulsivity in Internet-addictive disorders: a comparison with pathological gambling. *Acta Neuropsychiatrica*, *28*(02), 92–100. http://doi.org/10.1017/neu.2015.54

Zhou, Z., Zhu, H., Li, C., & Wang, J. (2014). Internet addictive individuals share impulsivity and executive dysfunction with alcohol-dependent patients. *Frontiers in Behavioral Neuroscience*, *8*(August), 288. http://doi.org/10.3389/fnbeh.2014.00288

# Description of cognitive domains and key outcome measures

Cognitive domains entering into the meta-analysis are briefly described below, including detailed of the preferred output measure(s). Sufficient suitable data were found for meta-analysis of the following cognitive domains (tasks): attentional inhibition (Stroop), motor inhibition (go/no go), pre-potent motor inhibition (stop-signal), decision-making (Cambridge Gambling Task, Iowa Gambling Task, Game of Dice, and Balloon Analogue Risk), working memory (Digit Span, Spatial Working Memory), and discounting. These domains were operationally defined as follows: motor inhibition, the ability to withhold not-already-triggered responses (Chamberlain & Sahakian, 2007); pre-potent motor inhibition, the ability to suppress already-triggered responses (Chamberlain & Sahakian, 2007); attentional inhibition, the ability to shift attention away from distracting environmental dimensions (MacLeod, 2015); decision-making, selection of optimal choices considering all available information (including risk & reward magnitudes, and probabilities of different outcomes) (Winstanley & Clark, 2016); working memory, ability to hold and manipulate information ‘on-line’ (Owen et al., 1999); and discounting, preference for larger more advantageous delayed rewards versus smaller more immediate rewards (Kirby & Finch, 2010).

### Stroop attentional inhibition

During the Stroop test, the presentation of incongruent stimuli (e.g. the word ‘GREEN’ in ‘red’ font) increases the executive processing burden for the brain and typically delays reaction times, compared to the response to congruent stimuli. Deficits have been demonstrated before in patients with substance misuse (Barch, Carter, & Cohen, 2004; Simon et al., 2002), among other disorders. Therefore, the preferred metric extracted from studies was the difference in reaction time between incongruent and congruent trials. Where this information was not available, the reaction time for incongruent trials was used.

### Motor Inhibitory Control

Inhibitory control is typically examined using go/no-go tasks and Stop-signal tasks. The former examines withholding responses in response to a certain cue, whereas the latter involves inhibition of already-triggered motor responses when a ‘stop-signal’ occurs. As such each of these two task types were analysed separately. Go/no-go tasks have shown validity as a measure of impulse control, and patients with attention-deficit hyperactivity disorder, or substance misuse have been shown to perform poorly compared to matched controls (Fillmore, 2003; Weafer, Milich, & Fillmore, 2011). For go/no-go tasks, inhibitory control is typically indexed by commission errors and this was the outcome measure of choice. The Stop-signal Task is a valid cognitive test to assess top-down inhibitory control performance (Verbruggen & Logan, 2008). Impulse control disorders, ADHD, compulsive spectrum disorders and substance misuse have been associated with deficits in motor response inhibition (Torregrossa, Quinn, & Taylor, 2008). The preferred metric used in the meta-analysis was the stop-signal reaction time, which is a measure of time taken to suppress a response that would normally be made.

### Decision-making

Decision-making is an executive function cognitive domain which can be assessed by a wide variety of cognitive performance tests. We included in the meta-analysis papers that used decision making tasks e.g. the Cambridge Gambling task (Chamberlain et al., 2017), the Iowa Gambling Task (Bechara, Damasio, Damasio, & Anderson, 1994), the Game of Dice Task (Brand, Recknor, Grabenhorst, & Bechara, 2007) and the balloon analogue risk task (BART) (Lejuez et al., 2002).

### Working memory

Working memory has been assessed using a spatial working memory task and an arithmetic working memory paradigm (digit span test). Working memory deficits have been demonstrated in patients with affective disorders (Sweeney, Kmiec, & Kupfer, 2000), among others. The metric used in the meta-analysis, where available was backward digit span (for the arithmetic paradigm) and total errors (for spatial working memory).

### Discounting

Discounting tasks examine whether participants prefer a smaller more immediate reward rather than a larger delayed reward. There were four studies in scope, however, due to limitations which are discussed in detail in the online supplement, the results presented separately (Supplementary Figures S1 & S2).
